# Host Plant Resistance to *Bemisia tabaci* to Control Damage Caused in Tomato Plants by the Emerging Crinivirus Tomato Chlorosis Virus

**DOI:** 10.3389/fpls.2020.585510

**Published:** 2020-10-14

**Authors:** Isabel M. Fortes, Rafael Fernández-Muñoz, Enrique Moriones

**Affiliations:** Instituto de Hortofruticultura Subtropical y Mediterránea “La Mayora”, Universidad de Málaga-Consejo Superior de Investigaciones Científicas (IHSM-UMA-CSIC), Estación Experimental “La Mayora”, Málaga, Spain

**Keywords:** tomato chlorosis virus, tomato yellow leaf curl virus, crinivirus, begomovirus, *Bemisia tabaci*, whitefly resistance, tomato

## Abstract

Tomato chlorosis virus (genus *Crinivirus*, family *Closteroviridae*) (ToCV) is rapidly emerging, causing increased damage to tomato production worldwide. The virus is transmitted in a semipersistent manner by several whitefly (Hemiptera: Aleyrodidae) species and is expanding its geographical and host ranges associated with the emergence of whiteflies of the *Bemisia tabaci* complex. Control is based essentially on intensive insecticide applications against the insect vector but is largely ineffective. No virus-resistant or tolerant commercial tomato cultivars are available. Recently, a *B. tabaci*-resistant tomato line based on the introgression of type IV leaf glandular trichomes and secretion of acylsucroses from the wild tomato *Solanum pimpinellifolium* was shown to effectively control the spread of tomato yellow leaf curl virus, a begomovirus (genus *Begomovirus*, family *Geminiviridae*) persistently transmitted by *B. tabaci*. As short acquisition and transmission periods are associated to the semipersistent transmission of ToCV, its possible control by means of the *B. tabaci*-resistant tomato could be compromised. Moreover, if the antixenosis effect of the resistance trait present in those tomato plants results in increased *B. tabaci* mobility, an increased ToCV spread might even occur. We demonstrated, however, that the use of acylsugar-producing *B. tabaci*-resistant tomatoes effectively controls ToCV spread compared to a near-isogenic line without type IV trichomes and acylsugar secretion. No increase in the primary ToCV spread is observed, and secondary spread could be reduced significantly decreasing the incidence of this virus. The possible use of host plant resistance to whiteflies to limit spread of ToCV opens up new alternatives for a more effective control of this virus to reduce the damage caused in tomato crops.

## Introduction

Plant virus infections represent a severe constraint to tomato (*Solanum lycopersicum* L.) production worldwide. Among them, emerging whitefly (Hemiptera: Aleyrodidae)-transmitted viruses such as the crinivirus (genus *Crinivirus*, family *Closteroviridae*) tomato chlorosis virus (ToCV) or begomoviruses (genus *Begomovirus*, family *Geminiviridae*) causing the tomato yellow leaf curl disease (tomato yellow leaf curl virus, TYLCV, the most widespread type), severely damage tomato crops worldwide (Hanssen et al., 2010; Rybicki, 2015; Rojas et al., 2018; Fiallo-Olivé and Navas-Castillo, 2019). Emergence of ToCV and TYLCV has been associated with the global spread of the whitefly *Bemisia tabaci* (Gennadius) in tropical and warm regions worldwide (Tzanetakis et al., 2013; Fereres, 2015; Gilbertson et al., 2015; Rojas et al., 2018; Fiallo-Olivé and Navas-Castillo, 2019). ToCV is transmitted in a semipersistent manner (short acquisition and inoculation periods) and TYLCV in a persistent manner (long acquisition and inoculation periods), respectively (Cohen and Antignus, 1994; Wintermantel, 2016; Fiallo-Olivé and Navas-Castillo, 2019).

Control of whitefly-transmitted viruses in tomato crops relies frequently on intensive chemical applications against the insect vector to reduce virus spread (Lapidot et al., 2014; Rojas et al., 2018; Fiallo-Olivé and Navas-Castillo, 2019). In fact, control of ToCV is mainly based in insecticide applications against whiteflies but has proven largely ineffective (Fiallo-Olivé and Navas-Castillo, 2019). Virus-resistant tomatoes are widely used commercially to effectively reduce the damage caused by TYLCV (Lapidot and Friedmann, 2002; Vidavski et al., 2008). However, although resistance of tomato plants to ToCV infection has been explored with sources from wild tomato relatives localized (García-Cano et al., 2010), no resistant/tolerant commercial tomato cultivar is yet available. Therefore, the recent report of a *B. tabaci*-resistant tomato line based on the introgression of type IV leaf glandular trichomes bred from the wild tomato *S. pimpinellifolium* useful to control the persistently transmitted begomovirus TYLCV (Rodríguez-López et al., 2011) offered new possibilities for the control of ToCV infections in tomato crops. Type IV trichomes in tomato are known to have acylsugars in their exudates which have been associated with negative effects on hemipteran pests (Simmons and Gurr, 2005). Nevertheless, the rapid acquisition and inoculation periods characteristic of semipersistent transmission of criniviruses, ToCV among others (Whitfield et al., 2015; Wintermantel, 2016), might compromise the control of this virus using *B. tabaci*-resistant tomatoes. Moreover, if the antixenosis effect of the resistance trait present in those acylsugar-producing tomato plants (Rodríguez-López et al., 2011) results in higher *B. tabaci* mobility, an increased ToCV spread might even occur. Therefore, the potential use of *B. tabaci*-resistant tomatoes to control ToCV spread is still an open question. Here, we provide evidence that support the use of *B. tabaci* resistance based on trichome production of acylsucroses as a management alternative to reduce ToCV spread during epidemics in tomato crops.

## Materials and Methods

### Tomato Plants

Two tomato lines were used in this work, the whitefly and virus susceptible tomato cv. Moneymaker and its near-isogenic genotype, the advanced backcross line ABL 14-8. The latter was generated from the initial cross *S. lycopersicum* cv. Moneymaker (without type IV leaf glandular trichomes) x *S. pimpinellifolium* accession TO-937 (with type IV leaf glandular trichomes, IHSM-CSIC germplasm collection) (Fernández-Muñoz et al., 2003). Three cycles of combined recurrent crosses toward “Moneymaker” and subsequent selfing steps with selection for high type-IV leaf glandular trichome density and acylsugar production traits were done followed by two additional final selfing steps. Seedlings were individually sown in plastic pots of 12 cm containing a mixture of 50% soil (54% sand, 24% silt, 22% clay), 30% horticultural substrate, 15% coconut-fiber substrate and 5% litonite. Until used, plants were grown within an insect-proof glasshouse under natural lighting with loose temperature control (22–27°C day, 17–20°C night) and supplied weekly with nutrient solution. Experiments were conducted taking into account the plant growth stage because significant difference in acylsucrose production between “Moneymaker” and ABL 14-8 is only achieved after plants reach the 10-leaf growth stage (Rodríguez-López et al., 2011).

### Virus Isolates, Whitefly Colony, and Virus Inoculation

ToCV isolate Pl-1-2 was used. This isolate was obtained from a naturally infected tomato plant collected during 1997 in Málaga (southern Spain) from a commercial tomato crop and maintained at IHSM in tomato cv. Moneymaker by periodic transmission with *B. tabaci* (García-Cano et al., 2010). The infectious clone of isolate [ES:Alm:Pep:99] of the Israel strain of TYLCV (hereafter, TYLCV), has been described elsewhere (Morilla et al., 2005). Virus-free *B. tabaci* (Mediterranean species) individuals were obtained from a colony originating from individuals collected during field visits in Málaga (Spain) and reared on melon (*Cucumis melo* L. cv. ANC42, IHSM seedbank collection) plants within wooden cages covered with insect-proof nets, in an insect-proof glasshouse with temperature control (22–27°C day and 17–20°C night) and light supplementation when needed.

ToCV-infected tomato plants were obtained by *B. tabaci*-mediated inoculation using viruliferous whiteflies. TYLCV-infected tomato plants were obtained either by *B. tabaci*-mediated inoculation using viruliferous whiteflies or by *Agrobacterium tumefaciens*-mediated inoculation (agroinoculation) using the infectious clone (see above) and the stem puncture method described by Monci et al. (2005). Plants were inoculated at the three-leaf growth stage. Mock inoculated control plants were obtained following the same inoculation procedures but using virus-free whiteflies or virus-free *A. tumefaciens*.

For *B. tabaci*-mediated inoculation, viruliferous whiteflies were obtained by mass feeding of virus-free *B. tabaci* adults (48-h acquisition access period, within insect-proof cages) on tomato cv. Moneymaker plants infected with ToCV or TYLCV 30 days before used for virus acquisition. For whitefly inoculation of individual plants, clip-on cages containing 25 viruliferous whiteflies were used on each test plant for a 48-h inoculation access period (IAP). Following IAP, the plants were sprayed with insecticide and maintained until used in an insect-proof glasshouse with temperature control (22–27°C day and 17–20°C night) and light supplementation when needed.

### Primary Spread Experiments

**FIGURE 1:**
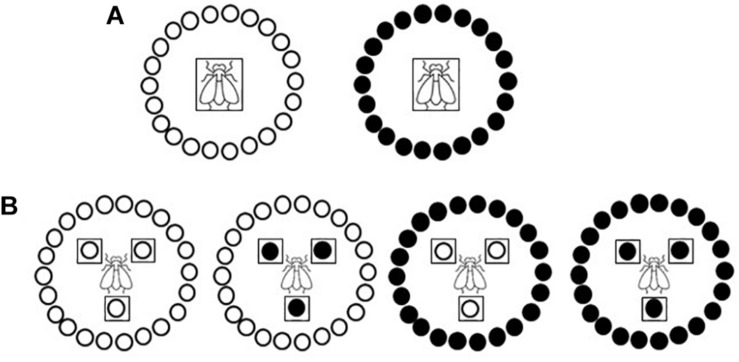
Schematic diagramof the primary and secondary spread experiments. Two near-isogenic tomato genotypes with or without type IV leaf glandular trichomes and acylsucrose secretions (ABL 14-8 and cv. Moneymaker, respectively) were used. **(A)** Primary spread to healthy “Moneymaker” or ABL 14-8 test plants (22 plants in a no-choice test) at 10-leaf growth stage using 15 viruliferous *Bemisia tabaci* Mediterranean (Med) species adult whiteflies per test plant and a 48-h feeding access period. **(B)** Secondary spread from 10-leaf growth stage “Moneymaker” or ABL 14-8 virus source plants to 10-leaf growth stage “Moneymaker” or ABL 14-8 healthy test plants (22 plants in a no-choice test) using 30 virus-free *B. tabaci* Med adult whiteflies per test plant and 96-h feeding access period. Virus source plants and viruliferous whiteflies are boxed; “Moneymaker” plants are represented with open circles, and ABL 14-8 plants with solid circles.

Primary spread, i.e., virus spread to healthy plants from external source viruliferous insect vectors (Campbell and Madden, 1990), was simulated in medium-scale field experiments conducted within whitefly-proof net (10 × 22 threads/cm^2^) walk-in structures (5 × 5 × 2 m) built within a tunnel net house at IHSM Experimental Station (Málaga, southern coastal Spain). Experiments were conducted by releasing 15 adult viruliferous whiteflies per test plant for a 48-h IAP. Viruliferous whiteflies were placed in the center of a circle (2 m diameter) of 22 healthy tomato test plants in a no-choice test design (Figure 1A). Experiments were conducted during summer with test plants at 10-leaf growth stage. Three independently repeated experiments were conducted with three replications per treatment in each one. After the IAP, plants were treated with insecticide and then transferred to an insect-proof glasshouse until analyzed. Type IV trichome density and acylsucrose accumulation on leaves were evaluated in assayed plants (see below). Virus presence was scored on the youngest newly emerged leaf of each test plant at weekly intervals until 28 days post inoculation (dpi) by tissue blot hybridization (see below). Propensity of the genotypes to be whitefly-infected was estimated by the percentage of infected plants. Data in the form of numbers of infected and non-infected plants were subjected to a generalized linear model analysis (Logit as the link function and Binomial as the underlying distribution) to perform statistical comparisons between the two genotypes by least-squares (LS) means test by using IBM SPSS Statistics for Windows, v. 26.0 (IBM Corp., Armonk, NY, United States).

### Secondary Spread Experiments

Secondary spread, i.e., virus spread from virus-infected source plants to healthy plants (Campbell and Madden, 1990), was simulated in medium-scale field experiments conducted within insect-proof net walk-in structures (see above). In each treatment three virus-infected source plants were used which were placed forming a triangle with 60 cm separation from each other, in the center of a circle (2 m diameter) of 22 healthy test plants (Figure 1B). Excess number of potential virus-infected source plants were prepared by agroinoculation (TYLCV) or by clip-on-cage viruliferous whitefly-mediated inoculation (ToCV) at the three-leaf stage. Then, the virus-infected source plants to be used in the experiments were selected for showing similar virus hybridization signals in apical leaves (see below). Virus-free *B. tabaci* adult individuals (30 whiteflies per test plant) were released in the center of the triangle of virus source plants and after 96 h, test plants were treated with insecticide and transferred to an insect-proof glasshouse until analyzed. Experiments were conducted during summer with test and virus-infected source plants at the 10-leaf growth stage. Two independently repeated experiments were performed with three replications per treatment in each one. Test plants were analyzed and statistical analyses of results were conducted as for primary spread experiments.

### Virus Detection by Molecular Hybridization

Presence of ToCV viral RNAs was analyzed in tomato plants by tissue blot hybridization. For this, freshly made cross-sections of petioles of the youngest newly emerged leaf of test plants were squash-blotted on positively charged nylon membranes (Roche Diagnostics GmbH, Mannheim, Germany). After blotting, nucleic acids were UV-cross-linked and hybridized with a ToCV-specific probe as described (García-Cano et al., 2006). For TYLCV detection, tissue blot hybridizations were also conducted as above using a TYLCV-specific probe (Monci et al., 2002). Although tissue-blotting is not a quantitative technique to determine virus accumulation, it was demonstrated to be useful in differentiating relative viral susceptibility among materials with different levels of resistance (Picó et al., 1999).

### Trichome Observation and Acylsucrose Accumulation Quantification

Leaf trichome density and targeted associated secretions were measured on leaflets of the third youngest leaf on 10-leaf growth stage plants. Type-IV trichome density was calculated following the indications by Alba et al. (2009). Previous analysis of TO-937 (the *S. pimpinellifolium* accession source of type IV leaf glandular trichomes introgressed in ABL 14-8) and the derived *S. lycopersicum* introgression line indicated that these produced sucrosyl esters. Epicuticular leaf acylsucroses were extracted and quantified according to Escobar-Bravo et al. (2016). To normalize the data and stabilize the variance, trichome density and acylsucrose production were Log (*x* + 1) transformed prior to analysis. Statistical differences between the means of trichome IV density and acylsucrose production in the two genotypes were analyzed by one-way ANOVA and the Fisher’s least significant difference (LSD) test by using IBM SPSS Statistics for Windows, Version 26.0 (IBM Corp., Armonk, NY, United States).

## Results

### Presence of Type-IV Leaf Glandular Trichome and Secretion of Acyl Sugars in ABL 14-8 Plants

**FIGURE 2:**
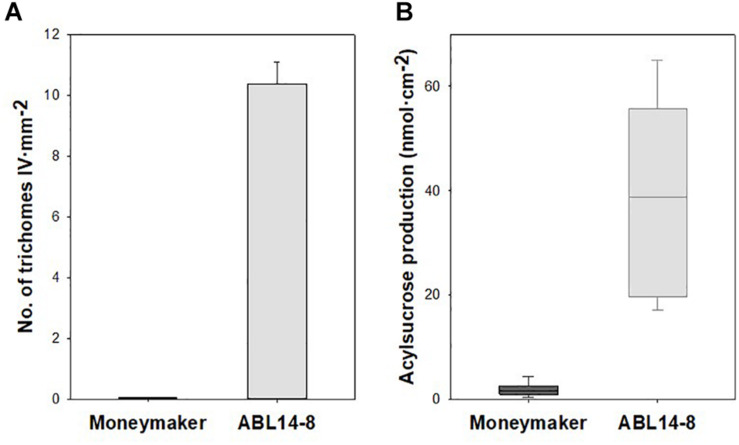
Type-IV leaf glandular trichome density and acylsucrose production in ABL 14-8 and “Moneymaker” near-isogenic tomato lines. **(A)**, mean values of type-IV leaf glandular trichome densities (+ SEM, *n* = 10) measured in “Moneymaker” and ABL 14-8 10-leaf growth stages plants. **(B)** Box-and-Whisker plots showing acylsucrose accumulation in young leaves of “Moneymaker” and ABL 14-8 plants (*n* = 10) at 10-leaf growth stage; the box represents the interquartile range, the horizontal line in the box shows the value of the median, and bars below and above the box mark the 10^th^ and 90^th^ percentiles. Plants were grown in warm (summer) conditions. Both type IV trichome densities and acylsucrose production were significantly different (*P* < 0.001) between the two lines.

Whereas type-I and type-VI glandular, and type-V non-glandular trichomes are present in both “Moneymaker” and its near-isogenic line ABL 14-8, the acylsucrose-producing type-IV leaf glandular trichomes, introgressed from *S. pimpinellifolium* accession TO-937, are absent in “Moneymaker” but densely cover the abaxial surface of ABL 14-8 leaves. As a result, in 10-leaf growth stage plants, significant differences were observed in the density of type IV trichomes and the acylsucrose production of “Moneymaker” and ABL 14-8 plants (Figure 2).

### Virus Susceptibility of “Moneymaker” and ABL 14-8

For a correct assessment of the effect of insect resistance on the spread of ToCV (and TYLCV control), the ABL 14-8 and “Moneymaker” genotypes to be compared should be equally susceptible to the virus. This was inspected and similar ToCV and TYLCV susceptibility was observed for both genotypes based on hybridization analyses (Supplementary Figure S1).

### No Increase of Primary ToCV Spread in ABL 14-8

**FIGURE 3:**
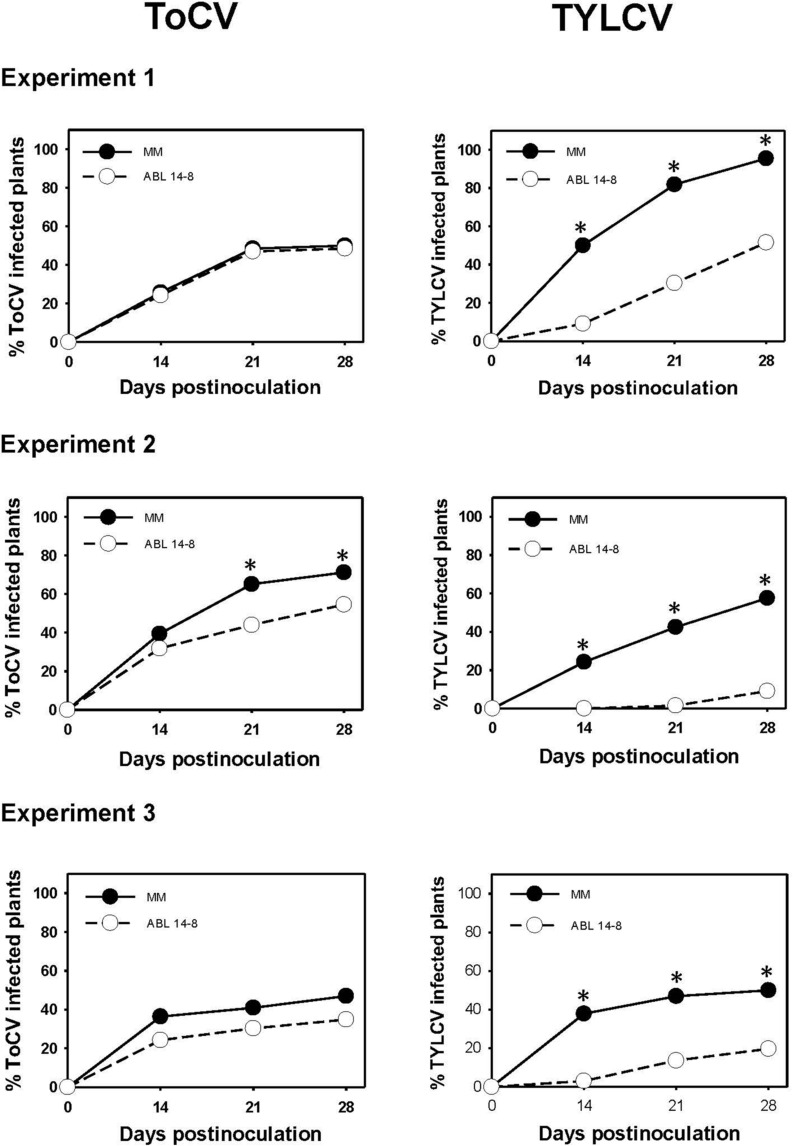
Primary spread of tomato chlorosis virus. Primary spread of tomato chlorosis virus (ToCV) and control tomato yellow leaf curl virus (TYLCV) to near isogenic healthy tomato test plants (22 plants in a no-choice test) with (ABL 14-8) or without (“Moneymaker,” MM) type IV leaf glandular trichomes and acylsucrose secretions at 10-leaf growth stage in medium-scale field experiments (three independently repeated experiments) conducted under warm-season conditions. ToCV and TYLCV transmission to test plants was measured several times after viruliferous whiteflies (15 *Bemisia tabaci* adult whiteflies per test plant) were given a 48-h feeding access period. Asterisk indicates significant differences in virus incidence between the two genotypes at a specific time (LS mean tests, *P* < 0.05).

The results of the medium-scale field experiments conducted to determine the possible effect of *B. tabaci*-resistance on primary ToCV spread are summarized in Figure 3. In no case was an increased ToCV spread observed owing to the presence of type IV glandular trichomes in ABL 14-8. As shown, in the three repeated experiments, ABL 14-8 plants exhibited an equal or lower propensity to be infected by ToCV compared to “Moneymaker,” even at statistically supported differences in some cases (Figure 3, Experiment 2). A significantly reduced primary spread of the persistently-transmitted begomovirus TYLCV in ABL 14-8 versus “Moneymaker” was observed in the control treatments included (Figure 3) in every experiment and as previously reported (Rodríguez-López et al., 2011). The latter confirmed the optimum expression and accumulation of acylsucroses from type IV leaf glandular trichomes present in the ABL 14-8 plants used. The lower primary spread propensity observed for ToCV in ABL 14-8 was less pronounced than that observed for TYLCV.

### Reduced Secondary ToCV Spread in ABL 14-8

**FIGURE 4:**
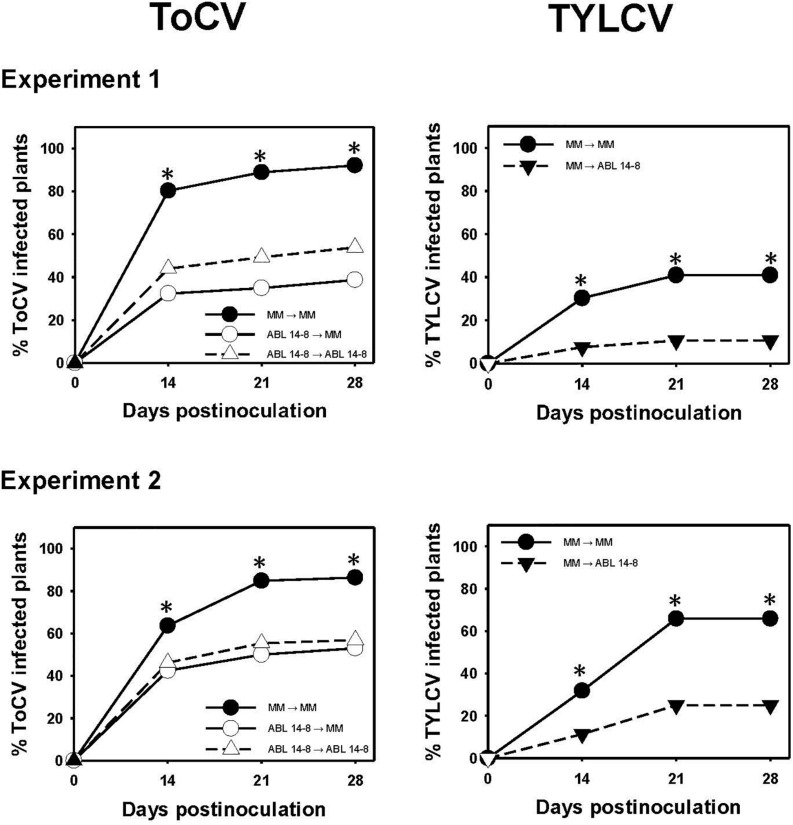
Secondary spread of tomato chlorosis virus. Secondary spread of tomato chlorosis virus (ToCV) and control tomato yellow leaf curl virus (TYLCV) from 10-leaf growth stage near isogenic tomato infected source plants with (ABL 14-8) or without (“Moneymaker,” MM) type IV leaf glandular trichomes and acylsucrose secretions to healthy ABL 14-8 or “Moneymaker” test plants (22 plants in a no-choice test) in medium-scale field experiments (two independently repeated experiments) conducted under warm-season conditions. Virus transmission to test plants was measured several times after virus-free whiteflies (30 *Bemisia tabaci* adult whiteflies per test plant) were given a 96-h feeding access period. Virus source (first) and test (second) plants are indicated in the figure legends. Asterisk indicates significant differences in virus incidence between the two genotypes at a specific time (LS mean tests, *P* < 0.05), which appeared only between the combination “Moneymaker” (source)-“Moneymaker” (test) and the other combinations.

Given that the previous results indicated that ABL 14-8 plants could be infected by ToCV, it was important to assess whether the *B. tabaci* resistance could help to limit the secondary spread of the virus from the infected source plants. As for the primary spread, in no case was an increased ToCV secondary spread observed owing to the presence of type IV glandular trichomes in ABL 14-8. As summarized in Figure 4, infected ABL 14-8 plants resulted in significantly less efficient virus sources for secondary ToCV spread at any time assessed compared to “Moneymaker.” About 20% to 40% less ToCV-incidence was detected at the end of the test period when ABL 14-8 was the virus source plant. No significant differences were observed for ABL 14-8 or “Moneymaker” as test plants (Figure 4). As expected from already reported studies (Rodríguez-López et al., 2011), significantly reduced secondary spread was also observed from ABL 14-8 source plants for the persistently-transmitted begomovirus TYLCV in the control treatments included (Figure 4). The latter confirmed the optimum expression and accumulation of acylsucroses from type IV leaf glandular trichomes present in the ABL 14-8 plants used. Therefore, the *B. tabaci* resistance present in ABL 14-8 was effective to impair secondary spread of the semipersistently transmitted ToCV.

## Discussion

Here a clue is given on the possibility to apply insect-resistant host plant to control a vectored plant virus. Control of ToCV infections in tomato crops is a challenge as yield losses of up to 50% of the tomato production have been reported (Mansilla-Córdova et al., 2018). In the absence of effective control measures the possibility to interfere with virus uptake and transmission (Dietzgen et al., 2016) was explored as an alternative management strategy. The results demonstrated that the use of the *B. tabaci*-resistance present in the ABL 14-8 tomato genotype based on type IV leaf glandular trichomes and acylsucrose exudates helps to impair ToCV spread which might be useful in controlling the damage caused by this virus.

The specific factors that determine ToCV transmission are complex, involving not only the virus and the insect vector but also the host plant and the environmental conditions. However, in addition to the nature of virus association with the vector, vector landing and probing on the plant as well as vector feeding patterns might condition the efficiency of virus transmission (Fereres and Moreno, 2009). Evidence is provided here supporting the idea that the presence of type IV leaf glandular trichomes and acylsucrose secretions in the tomato host can alter *B. tabaci* behavior in a way that can lead to changes in ToCV transmission. Alteration of virus transmission can then result in changes of virus spread dynamics (Jeger et al., 2004). No increase of primary ToCV spread was observed in ABL 14-8 in the experiments conducted in the present work. Moreover, a tendency to less efficient ToCV spread was observed in some cases. We had previously demonstrated that the *B. tabaci*-resistance traits present in ABL 14-8, in addition to deterring insect landing, affected insect feeding behavior (Rodríguez-López et al., 2011). The possibility of using insect deterrence to reduce virus-associated crop losses has been highlighted (Mutschler and Wintermantel, 2006). Then, the less efficient ToCV primary virus spread shown in the *B. tabaci*-resistant tomato genotype could have been the result of a restricted *B. tabaci* interaction with these plants owing to their reduced attractiveness to the insect landing (Rodríguez-López et al., 2011). However, the relevance of *B. tabaci* feeding behavior after landing on a plant and the possible effects on virus transmission has also been discussed (Peñalver-Cruz et al., 2020). ToCV is a phloem-restricted virus in tomato and its inoculation is mainly associated with effective stylet activities in phloem sieve elements (Prado Maluta et al., 2017). Thus, the tendency to reduced primary spread observed in ABL 14-8 might also have been the result of the longer non-probe periods and shorter salivation times in the phloem reported for *B. tabaci* in ABL 14-8 (Rodríguez-López et al., 2011) which may have contributed to a less efficient ToCV inoculation. Therefore, both the *B. tabaci* restricted landing and the altered phloem feeding behavior in ABL 14-8 might contribute to a less effective primary virus spread.

For secondary virus spread, both virus acquisition from virus-infected source plants and virus inoculation in healthy plants should occur (Campbell and Madden, 1990). Therefore, an impaired secondary spread is expected in the *B. tabaci*-resistant ABL 14-8 tomato plants, as was observed. On the one hand, the *B. tabaci* deterrence reported in ABL 14-8 (Rodríguez-López et al., 2011) may have affected both virus uptake and virus transmission which might have resulted in the significantly decreased ToCV secondary spread observed. On the other hand, the reported altered feeding behavior of *B. tabaci* in this tomato genotype may also have affected ToCV secondary spread. The combination of an impaired virus acquisition expected from the longer non-probe periods and lower number of phloematic ingestions reported for *B. tabaci* in ABL 14-8 (Rodríguez-López et al., 2011), and an impaired ToCV inoculation that may result from the less efficient *B. tabaci* phloem salivation in the resistant plants (Rodríguez-López et al., 2011; Prado Maluta et al., 2017) might also support a less efficient secondary spread. Therefore, as for primary spread, the sum of the effects of the *B. tabaci* restricted landing and the altered phloem feeding behavior in ABL 14-8 might have resulted in the less effective secondary virus spread observed.

Madden et al. (2000) have stressed that the effect of vector resistance on disease development can strongly depend on the virus transmission mode. ToCV is a semipersistently transmitted virus (Wintermantel, 2016), with short acquisition and transmission periods. If these periods were enough for virus spread in ABL 14-8 and the *B. tabaci*-resistance traits present in plants of this tomato genotype stimulate greater movement of the whiteflies, an increased ToCV spread might have been feared. In fact, increased spread of non-persistently transmitted viruses (very short acquisition and transmission periods) has been reported in insect vector resistant plants in which the virus can be transmitted during the insect’s short feeding probes for suitable feeding sites (Atiri et al., 1984). Also, a similar increased virus spread has been observed for non-persistently transmitted viruses with the use of some insecticides that agitate the insects and encourage movement to and feeding on greater numbers of plants before the time they die (Dent, 2001). The results obtained here, however, did not support an increased spread associated with the *B. tabaci*-resistance in ABL 14-8 which is strongly relevant for ToCV control purposes. In addition, a reduced secondary spread of ToCV was shown, mimicking the already reported response for the case of the persistently transmitted TYLCV (Rodríguez-López et al., 2011). Then, the effective reduced spread of the semipersistently transmitted ToCV obtained here by using the *B. tabaci*-resistant ABL 14-8 genotype might help to manage this virus in tomato crops. Future field testing can help in consolidating the results obtained here. Intensive tomato production mostly done under greenhouse conditions can be the perfect scenario for such field trials based on the results obtained. In these conditions, passive insect containment is conducted using screen nets in windows to minimize *B. tabaci* influx into the crop. In these conditions, the primary virus spread due to the continuous influx of viruliferous insects is mostly contained and secondary spread from initial virus foci is especially relevant. Conducting field tests under greenhouse conditions, might then allow the evaluation of the effect of the tomato plants ABL 14-8 on the reduction of secondary transmissions of ToCV, for which experimental data are robust. Moreover, if mixed ToCV-TYLCV infections occur during field trials, periodic monitoring of the exposed plants using molecular techniques will allow assessing single and mixed infections for an extended period. This can help to obtain more general conclusions about the benefits of the use of resistance to the vector, conferred by the glandular trichomes, to reduce the spread of these two viruses.

Management of viruses through alteration of vector efficiency as shown here for *B. tabaci* and ToCV is an interesting control alternative that might help to reduce dependence on intensive insecticide use (Zitter and Simons, 1980). This is especially important in cases in which no host-plant resistance to the virus is commercially available, as is the case for ToCV. Moreover, the use of insect-resistance in the host is also valuable for improving the management of *B. tabaci* as a pest, because it has been shown that insecticide pressure rapidly results in the development of resistant populations (Horowitz et al., 2020). As a result, the *B. tabaci* resistance described here would also be useful for supplementing *B. tabaci* control measures (Perring et al., 2018) in order to reduce the dependence on insecticide applications.

## Conclusion

In conclusion, the *B. tabaci*-resistance derived from the presence of type IV leaf glandular trichomes and acylsucrose production present in ABL 14-8 tomato plants significantly helps to reduce ToCV spread even though this tomato genotype is fully susceptible to the virus. This resistance might then be an interesting tool to be included in integrated management of ToCV epidemics. As ToCV-resistance sources have also been reported from tomato relatives (García-Cano et al., 2010) it may be expected that ToCV-resistance will soon be bred in commercial tomatoes. It has been shown that the use of vector resistant lines to reduce virus spread helps to exert minimal selection pressure on the virus to evolve more harmful strains (van den Bosch et al., 2006). Then, the alternative of combining *B. tabaci*-resistance with virus-resistance genes (gene pyramids) will offer future alternatives for a more effective, sustainable and durable control of ToCV (Gómez et al., 2009; Mundt, 2014).

## Data Availability Statement

All datasets presented in this study are included in the article/Supplementary Material.

## Author Contributions

EM conceived and developed the concept, supervised the experiments, and wrote the manuscript. RF-M contributed to the data analysis and interpretation, discussions, and writing of the manuscript. IF performed the experiments. All the authors contributed to the manuscript revision, read, and approved the final manuscript.

## Conflict of Interest

The authors declare that the research was conducted in the absence of any commercial or financial relationships that could be construed as a potential conflict of interest.

## Supplementary Material

Supplementary Figure 1 Accumulation of tomato chlorosis virus and tomato yellow leaf curl virus in isogenic tomato genotypes ABL 14-8 and “Moneymaker.” Molecular hybridization of squash blots of freshly cross-sectioned young leaves petioles performed at 30 days post-inoculation for nine plants of each of the “Moneymaker” or ABL 14-8 near-isogenic tomato lines inoculated by using tomato chlorosis virus (ToCV) **(A,B)** or tomato yellow leaf curl virus (TYLCV) **(D,E)** with clip-on-caged viruliferous whiteflies; short **(A,D)** and long **(B,E)** exposition of autoradiographs are shown. In order to prevent interference of the *Bemisia tabaci* resistance of ABL 14-8 in the assessment of the host-plant susceptibility to the viruses of the two nearly isogenic lines, the plants were inoculated at the three-leaf growth stage when acylsucrose production of type IV leaf glandular trichomes is still very low (Rodríguez-López et al., 2011). For monitoring of virus accumulation, one leaf petiole was tested per test plant performing two squash blots. Molecular hybridization was conducted with probes specific to ToCV or TYLCV. Numbers on top of the autoradiographs refer to the plant number. The two genotypes were equally susceptible to each virus in terms of both the number of infected plants and the estimation of viral accumulation done by densitometry measure of hybridization signals obtained in digitized imaging of autoradiographs. The comparison of the hybridization signals for the short exposition autoradiographs obtained from ToCV and TYLCV infected “Moneymaker” and ABL 14-8 plants is shown in **(C)** and **(F)**. respectively. Densitometry measurements were expressed as adjusted pixel densities calculated using Quantity One Software v 4.6.7 (VersaDoc MP 4000 Imaging System; Bio-Rad). Values were then represented in Box-and-Whisker plots and compared by One way ANOVA by using IBM SPSS Statistics for Windows, v. 26.0 (IBM Corp., Armonk, NY, United States). NS = non-significant differences.
