# Supplementary material for: Host Plant Resistance to Bemisia tabaci to Control Damage Caused in Tomato Plants by the Emerging Crinivirus Tomato Chlorosis Virus
Source: Front Plant Sci. 2020 Oct 14;11:585510. doi: 10.3389/fpls.2020.585510 (PMC7594678; doi:10.3389/fpls.2020.585510)
Supplement: Supplementary Figure 1 — Accumulation of tomato chlorosis virus and tomato yellow leaf curl virus in isogenic tomato genotypes ABL 14-8 and “Moneymaker.” Molecular hybridization of squash blots of freshly cross-sectioned young leaves petioles performed at 30 days post-inoculation for nine plants of each of the “Moneymaker” or ABL 14-8 near-isogenic tomato lines inoculated by using tomato chlorosis virus (ToCV) (A,B) or tomato yellow leaf curl virus (TYLCV) (D,E) with clip-on-caged viruliferous whiteflies; short (A,D) and long (B,E) exposition of autoradiographs are shown. In order to prevent interference of the Bemisia tabaci resistance of ABL 14-8 in the assessment of the host-plant susceptibility to the viruses of the two nearly isogenic lines, the plants were inoculated at the three-leaf growth stage when acylsucrose production of type IV leaf glandular trichomes is still very low (Rodríguez-López et al., 2011). For monitoring of virus accumulation, one leaf petiole was tested per test plant performing two squash blots. Molecular hybridization was conducted with probes specific to ToCV or TYLCV. Numbers on top of the autoradiographs refer to the plant number. The two genotypes were equally susceptible to each virus in terms of both the number of infected plants and the estimation of viral accumulation done by densitometry measure of hybridization signals obtained in digitized imaging of autoradiographs. The comparison of the hybridization signals for the short exposition autoradiographs obtained from ToCV and TYLCV infected “Moneymaker” and ABL 14-8 plants is shown in (C) and (F). respectively. Densitometry measurements were expressed as adjusted pixel densities calculated using Quantity One Software v 4.6.7 (VersaDoc MP 4000 Imaging System; Bio-Rad). Values were then represented in Box-and-Whisker plots and compared by One way ANOVA by using IBM SPSS Statistics for Windows, v. 26.0 (IBM Corp., Armonk, NY, United States). NS = non-significant differences. [file Image_1.pdf]

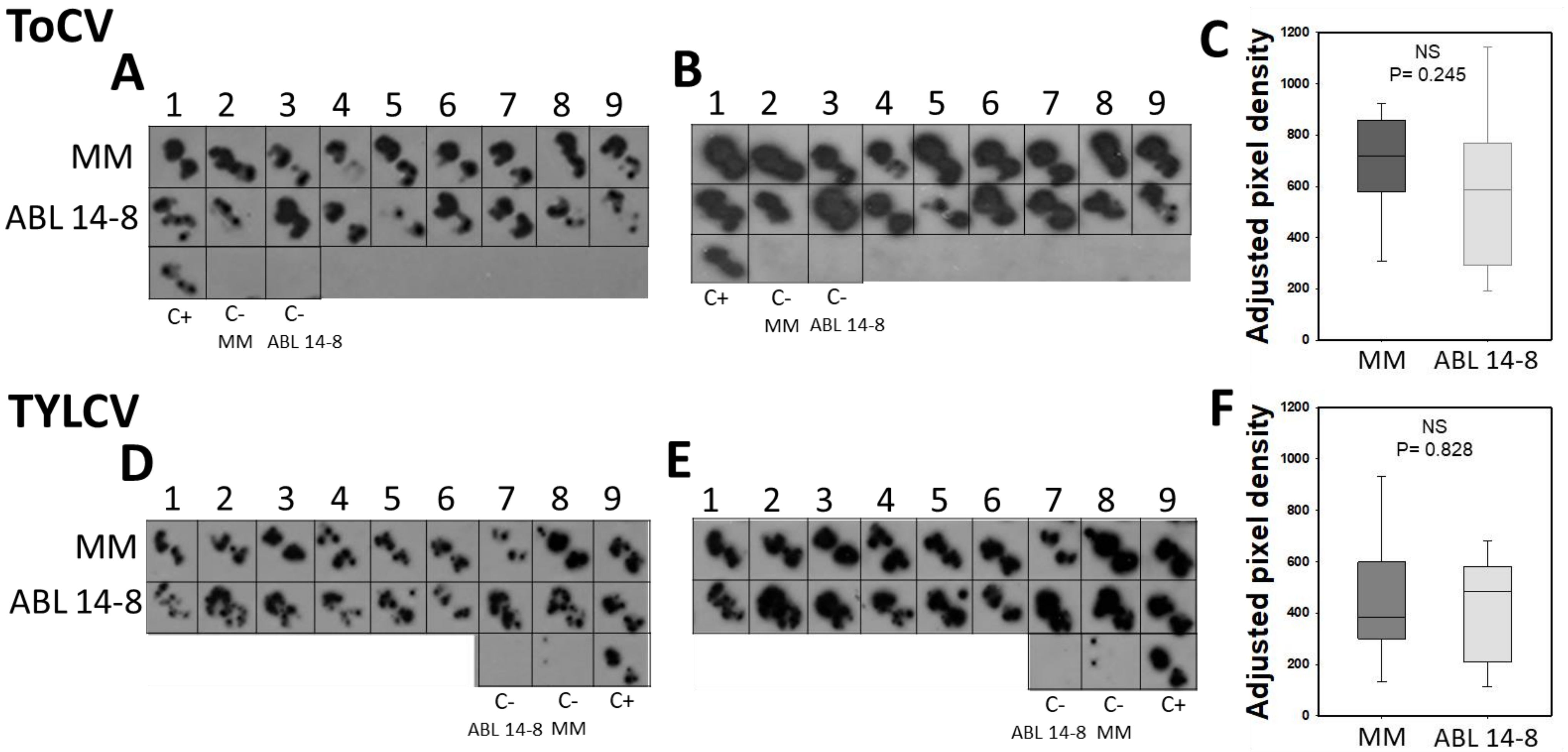

Supplmentary Figure S1. Accumulation of tomato chlorosis virus and tomato yellow leaf curl virus in isogenic tomato genotypes ABL 14-8 and 'Moneymaker'. Molecular hybridization of squash blots of freshly cross-sectioned young leaves petioles performed at 30 days post-inoculation for 9 plants of each of the 'Moneymaker' or ABL 14-8 near-isogenic tomato lines inoculated by using tomato chlorosis virus (ToCV) **(A, B)** or tomato yellow leaf curl virus (TYLCV) **(D, E)** with clip-on-caged viruliferous whiteflies; short **(A, D)** and long **(B, E)** exposition of autoradiographs are shown. In order to prevent interference of the *Bemisia tabaci* resistance of ABL 14-8 in the assessment of the host-plant susceptibility to the viruses of the two nearly-isogenic lines, the plants were inoculated at the three-leaf growth stage when acylsucrose production of type IV leaf glandular trichomes is still very low (Rodríguez-López et al., 2011). For monitoring of virus accumulation, one leaf petiole was tested per test plant performing two squash blots. Molecular hybridization was conducted with probes specific to ToCV or TYLCV. Numbers on top of the autoradiographs refer to the plant number. The two genotypes were equally susceptible to each virus in terms of both the number of infected plants and the estimation of viral accumulation done by densitometry measure of hybridization signals obtained in digitized imaging of autoradiographs. The comparison of the hybridization signals for the short exposition autoradiographs obtained from ToCV and TYLCV infected 'Moneymaker' and ABL 14-8 plants is shown in C and F. respectively. Densitometry measurements were expressed as adjusted pixel densities calculated using Quantity One Software v 4.6.7 (VersaDoc MP 4000 Imaging System; BioRad). Values were then represented in Box-and-Whisker plots and compared by One way ANOVA by using IBM SPSS Statistics for Windows, v. 26.0 (IBM Corp., Armonk, NY). NS = non-significant differences.
